# An Unusual Presentation of Giant Fibroepithelial Polyp of the Vagina

**DOI:** 10.30699/IJP.2021.522689.2566

**Published:** 2021-06-06

**Authors:** Azar Daneshpajooh, Mahboubeh Mirzaei, Maryam Iranpour

**Affiliations:** 1 Department of Urology, Shahid Bahonar Hospital, Kerman University of Medical Sciences, Kerman, Iran; 2 Department of Pathology, Pathology and Stem Cell Research Center, Faculty of Medicine, Kerman University ofMedical Sciences, Kerman, Iran

**Keywords:** benign tumor, Fibroepithelial Polyps, Vaginal polyps

## Abstract

Fibroepithelial polyps of the vagina (FEPV) are rare entities which normally manifest as one or more painless polyps sometimes with symptoms such as bleeding, vaginal discharge, and discomfort regarding the size of the mass. Despite their benign nature, they can be confused with other vaginal tumors due to their abnormal histology. In this report, we present a case of a 44-year-old woman with a giant pedunculated and symptomatic polyp of the vagina with anterior vaginal wall prolapse. The treatment method included a simple local excision of the polyp and anterior vaginal compartment repair. Histopathological examination revealed a polypoid lesion covered by squamous epithelium containing a central fibrovascular core without atypia. The patient experienced an uneventful postoperative recovery, with no complication, which implies that surgery is the most effective modality for managing such tumors.

## Introduction

Benign or malignant vaginal neoplasms are rare. Evidence shows that neoplasms developing in other sites within the genital tract may also be observed in the vagina. Most vaginal tumors do not show any symptoms until they reach a significant size. Pressure sensation, dyspareunia, vaginal or urethral obstruction, and vaginal bleeding may be among the symptoms and signs (1).

Fibroepithelial polyps of the vagina (FEPV) are rare disorders, which normally manifest as one or more painless polyps. Symptoms, such as bleeding, vaginal discharge, and discomfort, may also be related to FEPV according to the size of the mass (2-4). Despite its benign nature, FEPV can be misidentified as malignant connective tissue lesions due to the abnormal histology it has. Sarcoma botryoides, rhabdomyosarcoma, and mixed mesodermal tumor make up the differential diagnoses for FEPV (5).

Herein, we report a case of FEPV, coexisting with advanced anterior vaginal wall prolapse.

## Case Presentation

The patient was a 44-year-old woman with a vaginal polyp, referred to the female urology clinic of Kerman University of Medical Sciences, Kerman, Iran. She presented with symptomatic anterior vaginal wall prolapse and a pedunculated mass, arising from the vagina. She had experienced the sensation of a vaginal mass, pelvic pressure, and sexual difficulty for six months. Moreover, she had presented with storage lower urinary tract symptoms (LUTS), such as dysuria, daytime urinary frequency, and urgency over the past several months. She also reported occasional mixed urinary incontinence.

According to the patient’s obstetric history, she was a premenopausal woman with a history of five pregnancies and four deliveries (three via normal vaginal deliveries and one through cesarean section), with the last one occurring eight years ago. She had been using oral contraceptive pills irregularly in the past 12 years and had a history of an ectopic pregnancy, leading to surgery and removal of an ovary. Her physical examination was within normal limit, and her body mass index (BMI) was 29 kg/m^2^.

Pelvic Examination

**Fig 1:**
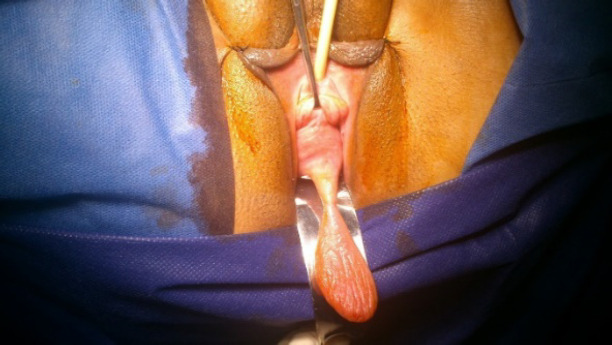
The patient’s pedunculated vaginal polyp

A pelvic examination was conducted with the patient in the lithotomy position. The genitalia were inspected, and no vulvar abnormality was observed. A retractor was used to depress the posterior vagina for visualizing the anterior vagina. The pelvic examination revealed a mobile mass, protruding from the vagina and attached to the anterior vaginal wall with its stalk. The surface of the mass indicated several folds, without bleeding or discharge. On palpation, the mass was not tender and had a firm consistency (Figure 1). In the vaginal examination with a speculum, the patient showed stage 3 cystocele, based on the Pelvic Organ Prolapse Quantification (POPQ) system, and the cough stress test was negative. Also, the cervix was healthy, and the Pap smear was normal. 

Diagnostic Tests

Urinalysis and culture were performed to evaluate urinary tract infections. Abdominopelvic ultrasonography was normal. Also, a multi-channel urodynamic test was performed to determine the cause of incontinence. During filling cystometry, the prolapse was reduced with a pessary. The filling phase showed normal bladder capacity and compliance. Also, low-amplitude detrusor overactivity and negative stress test were reported in filling cystometry. Moreover, the pressure flow study revealed a normal flow rate and detrusor pressure; the post-void residual volume was 40 cc. 

Surgical Technique

**Fig 2:**
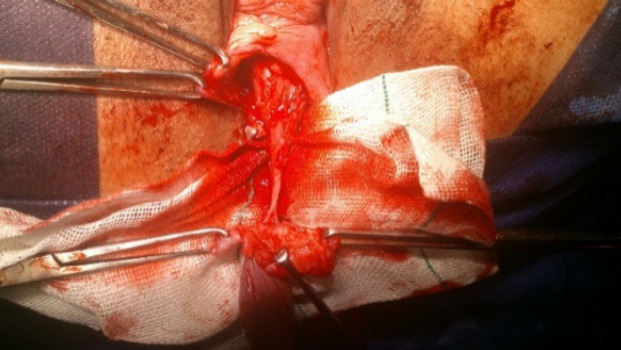
Excision of the polyp

The patient was placed in the lithotomy position, and cystoscopy was performed to examine the bladder and urethra. Next, a 16-Fr urethral Foley catheter was passed into the urethra. An inverted-U incision was made on the anterior vaginal wall, and the anterior vaginal mucosa was dissected off the underlying vesicovaginal septum by sharp dissection. Next, complete excision of the vaginal polyp was carried out, the neck of the polyp was dissected from the bladder (Figure 2), and mobilization of the bladder base was completed (intact bladder). Subsequently, vaginal mucosa was dissected laterally as much as possible up to the inferior pubic ramus. Afterward, hemostasis was secured, and the pubocervical fascia was reconstructed (from the upper to the lower part) with an interrupted suture. Finally, a delayed absorbable ‘00’ suture material was used, and the anterior vaginal mucosa was closed. 

Pathology Results

**Fig. 3:**
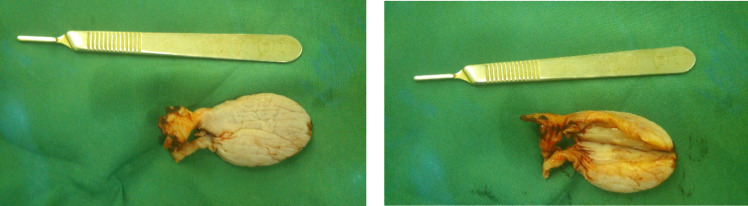
Excised vaginal polyp

**Fig 4:**
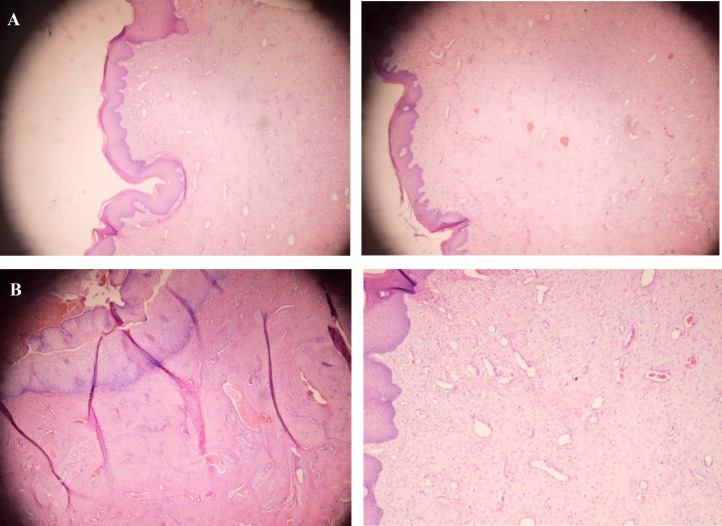
a) A polypoid lesion consisting of a squamous epithelium (H&E staining at 40× magnification), and b) a fibrovascular core with abundant blood vessels

Macroscopically, the specimen was a well-defined, pedunculated, skin colored polypoid lesion (7×3 cm) (Figure 3). Cut sections indicated a creamy area without necrosis. Embed: 60%, SOS:15 P / 9 Block (60% of the polyp has been histologically sectioned and reviewed). The specimen was fixed with 10% neutral formalin solution, and 6-µm thickness slides were prepared and stained via Haemotoxylin and Eosin (H&E) staining. Microsco-pically, the lesion was covered with a squamous epi-thelium with hypocellular edematous stroma (Figure 4a). Also, its central fibrovascular core consisted of loose hyper-vascular connective tissue with collagen bundles, and there was no atypia or necrosis (Figure 4b).

## Discussion

Neoplasms of the vagina are uncommon entities. The presentations of these neoplasms vary and may include pressure sensation, dyspareunia, urogenital obstruction, tissue protruding from the vagina, abdominal pain, and bloody discharge (6). FEPV, as a rare and usually asymptomatic tumor, may be small and multiple. Also, FEPV is described as a rare benign soft tissue tumor, occurring in the vulvovaginal area. It probably initiates from subepithelial stromal cells or subepithelial mesen-chymal cells in the vulvovaginal region from the endocervix to the vulva. It is observed commonly in the vagina, then the vulva and the cervix, and scarcely in extragenital sites (2, 3, 7).

FEPV is commonly observed in pregnant women, while it rarely occurs before menarche or after menopause, during pregnancy, the lesion may become enlarged, edematous, and abnormal in appearance (8). Histologically, FEPV consists of a squamous epithelial surface with a fibrovascular stalk and edematous stroma (9, 10). The etiology of these polyps may involve a granulation tissue response to some local injury to the vaginal mucosa. Delayed differentiation of myo-fibroblastic stromal cells may explain why the granulation tissue sometimes fails to contract properly and turns into polyps (10). However, in pregnancy, hormonal factors may modulate the growth of FEPV. In this regard, Hartmann CA *et al.*, after performing immunohistochemistry, reported that FEPV expressed vimentin, desmin, estrogen receptors, and progesterone receptors, indicating its hormone-dependent nature (11).

The pathogenesis of FEPV has not been explained properly. Nevertheless, a strong relationship with hormonal stimulation has been put forward, as it repeatedly occurs in pregnancy and regresses after delivery. It is also linked with hormone replacement therapy or tamoxifen treatment, and FEPV stromal cells react to estrogen receptors (12). Histopathologic studies can confirm the diagnosis of FEPV. Other terminologies for FEPV in the literature include cellular pseudosarcomatous, fibroepithelial stromal polyp, polyposis vaginalis, and pseudosarcoma botryoide (15). 

Microscopically, fibroepithelial stromal polyp consists of three components, including a central fibrovascular core, stroma with pedunculated or polypoid proliferation, and an overlying squamous epithelium. The stromal cells range from spindle to stellate-shaped cells, with some multinucleated forms; however, they generally have bland nuclear features. The distribution of these cells is variable but characteristic. In addition, the stellate and multinucleated forms tend to aggregate along the stromal-epithelial junction and around blood vessels of the central fibrovascular core. The squamous epithelium lying on the stromal proliferation is normal to hyperplastic. Moreover, the stromal cells of a fibroepithelial stromal polyp invariably react to desmin, estrogen receptors, progesterone receptors, and sometimes smooth muscle actins (16).

Treatment of FEPV involves simple local excision, and its recurrence is uncommon in patients (17, 18). In pregnant women, surgery can be performed after pregnancy when vaginal vascularity has returned to normal (19). In this regard, Nucci MR *et al.*, in a study on 65 cases of FEPV, stated that knowledge of the histopathological spectrum of these lesions is a key factor for their accurate diagnosis and preventing potential overtreatment. 

Overall, FEPV is recognized as a benign disorder, with no reported destructive local recurrence or metastasis, unless the resection margin is positive (20). In the present case, FEPV co-occurred with anterior vaginal wall prolapse, which is not a common presentation. Tissue trauma, caused by advanced cystocele and irregular use of oral contraceptive pills, could play a role in the pathophysiology of the polyp. Following preoperative assessments, including physical examination, FEPV was treated by excision. The cystocele was repaired, and the tumor was successfully removed by resection, resulting in the complete relief of the symptoms. The patient went through an uneventful postoperative recovery, and succeeding complications, such as infection, urinary incontinence, and dyspareunia, were not observed.

## Conclusion

FEPV is defined as a mucosal polypoid lesion with a connective tissue core, covered by a benign squamous epithelium. We described a rare case of concomitant FEPV and anterior vaginal wall prolapse in a 44-year-old woman. She experienced an uneventful postoperative recovery, with no complication, which implies that surgery is the most effective recourse for managing such tumors.
